# Role of LytF and AtlS in eDNA Release by *Streptococcus gordonii*

**DOI:** 10.1371/journal.pone.0062339

**Published:** 2013-04-24

**Authors:** Yifan Xu, Jens Kreth

**Affiliations:** 1 Department of Microbiology and Immunology, University of Oklahoma Health Sciences Center, Oklahoma City, Oklahoma, United States of America; 2 College of Dentistry, University of Oklahoma Health Sciences Center, Oklahoma City, Oklahoma, United States of America; 3 Department of Surgical Oncology, The First Hospital of China Medical University, Shenyang, PR China; University of Kansas Medical Center, United States of America

## Abstract

Extracellular DNA (eDNA) is an important component of the biofilm matrix produced by many bacteria. In general, the release of eDNA is associated with the activity of muralytic enzymes leading to obvious cell lysis. In the Gram-positive oral commensal *Streptococcus gordonii*, eDNA release is dependent on pyruvate oxidase generated hydrogen peroxide (H_2_O_2_). Addition of H_2_O_2_ to cells grown under conditions non-permissive for H_2_O_2_ production causes eDNA release. Furthermore, eDNA release is maximal under aerobic growth conditions known to induce pyruvate oxidase gene expression and H_2_O_2_ production. Obvious cell lysis, however, does not occur. Two enzymes have been recently associated with eDNA release in *S. gordonii*. The autolysin AtlS and the competence regulated murein hydrolase LytF. In the present report, we investigated the role of both proteins in the H_2_O_2_ dependent eDNA release process. Single and double mutants in the respective genes for LytF and AtlS released less eDNA under normal growth conditions, but the AtlS mutant was still inducible for eDNA release by external H_2_O_2_. Moreover, we showed that the AtlS mutation interfered with the ability of *S. gordonii* to produce eDNA release inducing amounts of H_2_O_2_. Our data support a role of LytF in the H_2_O_2_ eDNA dependent release of *S. gordonii* as part of the competence stress pathway responding to oxidative stress.

## Introduction

The biofilm developmental process requires the release of extracellular polymeric substances (EPS) by the biofilm forming community [1], [2]. The EPS is commonly composed of protein, polysaccharides, lipids and extracellular DNA (eDNA) [3], [4]. The presence of eDNA during the developmental process is important [5], since treatment of developing or preformed biofilms with DNA degrading enzymes disrupts the biofilm structure and stability [6], [7], [8]. In general, eDNA found in the biofilm EPS seems to be of microbial chromosomal origin [9], [10], [11]. Investigations on the integrity of the eDNA revealed largely intact DNA still carrying genomic information, which is further supported by the observation that eDNA is also a source for horizontal gene transfer [12], [13]. A recent detailed oligonucleotide array based study using a non-domesticated *Bacillus subtilis* strain showed that the biofilm recovered eDNA includes the whole genome without specific gene preferences [14]. Earlier studies with other bacterial species showed that randomly selected genes on different chromosomal locations are present in eDNA [10] suggesting that chromosomal DNA is released to serve as eDNA during biofilm development.

Different release/production mechanisms for eDNA of chromosomal origin seem to exist. For example, the bacteriolytic dependency of eDNA release was demonstrated for several species and is ultimately linked to bacterial cell death [11], [15], [16]. The regulatory relationship between eDNA release and cell death has been studied in detail in Gram-positive *Staphylococcus aureus* [17], [18], [19]. The Staphylococcal Cid/Lrg system encodes proteins analogous to the bacteriophage-encoded holins and antiholins. Initial studies suggest that the LrgA and CidA proteins function in similar mechanisms as the holins/antiholins in *S. aureus*, ultimately activating murein hydrolases leading to bacterial cell lysis [17], [18], [19]. Homologs of Cid/Lrg can be found in several species, including cariogenic *Streptococcus mutans*. Interestingly, the Cid/Lrg system in *S. mutans* is involved in several other cellular processes including competence development and oxidative stress tolerance, suggesting a connection between general stress and lysis dependent eDNA release in oral streptococci [20], [21]. In the opportunistic pathogen *Pseudomonas aeruginosa*, phage inductions in biofilms are implicated in the release of DNA as a result of phage mediated cell lysis [11], [22]. Other examples of lysis dependent eDNA release are the autolysin AtlE dependent eDNA release in *Staphylococcus epidermidis* [23] and the gelatinase GelE and serine protease SprE dependent eDNA release in *Enterococcus faecalis* [16].

An alternative to the cell-lysis dependent eDNA release mechanisms has been suggested in two recent studies in *E. faecalis* [24] and *B. subtilis* [14] where a lysis-independent eDNA release mechanism has been proposed. In *E. faecalis* well-defined structures of eDNA were observed supporting early biofilm development. However, no intracellular components indicative of cell lysis could be detected in cell free supernatants during the early biofilm developmental stage. Furthermore, cells implicated in the release of eDNA had an active membrane potential excluding a connection with bacterial cell death [24]. *B. subtilis* on the other hand has a mechanism to release eDNA in the late exponential phase. The authors of this study confirmed lysis-independence genetically by constructing several mutant strains with genes involved in bacterial lysis showing they are not reduced in the eDNA release. The release process appeared to be regulated by the *B. subtilis* early competence genes [14].

*Streptococcus gordonii* belongs to the group of early oral biofilm formers and it’s presence is critical for subsequent biofilm development since it provides attachment sites for other species [25]. *S. gordonii* as well as several other oral streptococci are known for their ability to produce competitive amounts of hydrogen peroxide (H_2_O_2_) during aerobic growth [26]. H_2_O_2_ production inhibits growth of competing species, but also induces the release of eDNA [10], [27], [28]. Furthermore, H_2_O_2_ has been demonstrated as the sole agent responsible for triggering the release process. The addition of H_2_O_2_ to *S. gordonii* grown under non-H_2_O_2_-producing conditions during static growth induces eDNA release, but no detectable autolysis [12]. Involvement of bacteriolytic enzymes in the eDNA release of *S. gordonii*, however, has been shown by two recent studies [13], [29]. Deletion of the autolysin AtlS causes a major decrease in eDNA release [29]. In addition, inactivation of the competence dependent murein hydrolase LytF reduced the gene transfer in a co-culture of competent *S. gordonii* with the LytF mutant about 100 fold. However, direct eDNA concentrations were not determined [13].

The present report presents a further characterization of the role of LytF and AtlS in the H_2_O_2_ dependent eDNA release of *S. gordonii*.

## Materials and Methods

### Bacterial Species and Culture Conditions

All *S. gordonii* strains used in this study listed in Tab. 1 were routinely grown aerobically (5% CO_2_) at 37°C in BHI (Brain Heart Infusion; Difco, Sparks, MD) unless otherwise stated. For antibiotic selection, cultures were supplemented with the following antibiotics: erythromycin at 5 µg ml^−1^ and kanamycin at 300 µg ml^−1^.

**Table 1:** Strains and oligonucleotides used in this study.

Strain	Relevant characteristics	Reference
DL1	Wild-type *S. gordonii*	[29]
DL1 AtlS	*atlS*; Kan^r^	[29]
DL1 AtlS-1	DL1 *AtlS*; *kan* resistance cassette replacedwith *erm* resistance cassette; Erm^r^	This study
DL1 LytF	Transformation of chrom. DNA from strain SGH24[13] carrying a *lytF* deletion, Kan^r^	This study
DL1 AtlS/LytF	DL1 AtlS-1 transformed with chrom. DNA fromstrain SGH24 [13] carrying a *lytF* deletion, Kan^r^, Erm^r^	This study
Oligonucleotides	Sequence	Purpose
gyrA RT-F	CCAAACCTTTTGGTCAATGG	Real time RT-PCR
gyrA RT-R	CCCAGGCAAAACTTCCATAA	Real time RT-PCR
16S rRNA Sg-F	AAGGAACGCGAAGAACCTTA	Real Time PCR
16S rRNA Sg-R	GTCTCGCTAGAGTGCCCAAC	Real Time PCR
spxB RT-F	GGATGCTTTGGCTGAAGAC	Real time RT-PCR
spxB RT-R	GGACCACCTGAACCTACTG	Real time RT-PCR
AtlS up-F	GAAATCCTGCGCAATAAAGC	AtlS K.O.
AtlS up-R	ATCAAACAAATTTTGGGCCCGGTGAA	AtlS K.O.
AtlS down-F	ATTCTATGAGTCGCTGCCGACTCCAA	AtlS K.O.
AtlS down –R	CATGCAGACATTATAGCA	AtlS K.O.
ermAM-F	CCGGGCCAAAAATTTGTTTGAT	AtlS K.O.
ermAM-F	CCGGGCCAAAAATTTGTTTGAT	AtlS K.O.
lytF RT-F	TTAATGGCCAAGACGACCTC	Real time RT-PCR
lytF RT-R	TACTTTCCCGCCAGATTGTC	Real time RT-PCR
Sg-ldh-up-F	GAAGGAGATGTTTAGAGAATGAC	ren-reporter
Sg-ldh-up-R-ren	TCTCTAAACATCTCCTTCTTAGTTTTTAGATGCTGCTTGGAATT	ren-reporter
Sg-ren-F	GAAGGAGATGTTTAGAGAATGACTTCGAAAGTTTATGATCCAG	ren-reporter
Sg-ren-R	CAAACAAATTTTGGGCCCGGTTATTGTTCATTTTTGAGAACTCGC	ren-reporter
Sg-ren-erm-F	GCGAGTTCTCAAAAATGAACAATAACCGGGCCCAAAATTTGTTTG	ren-reporter
Sg-ren-erm-R	CTCCTTTAATAAGGAGATGTTTTTATAAAGTCGGCAGCGACTCATAG	ren-reporter
Sg-ldh-down-F	CTATGAGTCGCTGCCGACTTTATAAAAACATCTCCTTATTAAAGGAG	ren-reporter
Sg-ldh-down-R	CCAAAAGATGTCTTGTCAGTTGG	ren-reporter

### Growth Kinetics

The growth of static wild type and mutant strains was monitored using a Bioscreen C analyzer version 2.4 (Oy Growth Curves AB Ltd., Finland), which measures the turbidity in multiple cultures in parallel for static cultures. Growth kinetics were monitored at 37°C in BHI in 20 min intervals. To measure growth under aerobic conditions with maximal H_2_O_2_ production, cells were grown in 15 ml screw cap tubes with a starting volume of 10 ml. The tubes were placed on a rocking table, promoting horizontal movement (Barnstead Thermolyne Vari-Mix), at 20 rpm and incubated at 37°C. Bacterial cell density was determined with a Genesys 20 spectrophotometer (Thermo Spectronic) at A_600 nm_.

### DNA Manipulations

Standard recombinant DNA manipulations were used [30]. PCR was performed with a G-Storm GS1 thermocycler (GeneTechnologies; Essex, UK) according to the manufacturer’s protocol. Phusion^®^ DNA polymerase was obtained from New England Biolab. Oligonucleotides (Tab. 1) were designed using sequence data obtained from the Los Alamos National Laboratory Oral Pathogens Sequence Database (http://www.oralgen.lanl.gov) and synthesized by Integrated DNA Technologies (Coralville, IA).

### Construction of Mutant Strains

The *lytF* mutant strain was constructed by transforming chromosomal DNA from strain SGH24 [13] carrying a *lytF* deletion using a transformation protocol reported earlier [31]. To construct a double AtlS/LytF K.O. mutant, the AtlS mutant was chosen to replace the *kan* antibiotic cassette with an *ermAM* cassette for compatibility with the *lytF* K.O. Replacement of the antibiotic cassette was done via double-crossover homologous recombination using a overlap PCR strategy. To generate the overlap PCR constructs, two fragments corresponding to around 500 bp of the upstream and downstream sequences of *atlS* were amplified by PCR, using Phusion^®^ DNA polymerase with the oligonucleotides AtlS up-F/AtlS up-R and AtlS down-F/AtlS down-R. Each of the oligonucleotides listed in Tab.1 as up-R and down-F incorporated 25 bases complementary to the erythromycin resistance cassette, *ermAM* [32]. The erythromycin resistance cassette *ermAM* was amplified by PCR using the primers ermAM F and ermAM R as described before [31]. All three PCR amplicons were purified with the QIAGEN PCR purification kit and mixed in a 1∶1:1 ratio. The mixture served as a template for a second round PCR with the appropriate up F and down R primers. The resulting PCR amplicons were transformed into DL1 AtlS to generate the deletion mutant DL1 AtlS-1. To create the double mutant, the LytF mutation was transformed into DL1 AtlS-1 as described above.

### Construction of Renilla Bioluminescent Reporter-strain and Renilla Assay

The renilla reporter strain was constructed via a four-piece overlapping PCR ligation strategy similar to the strategy described above. The renilla gene was set under the control of the *ldh* (lactate dehydrogenase) promoter from *S. gordonii*. The renilla gene was inserted downstream of the *ldh* stop codon to leave the *ldh* gene intact. Briefly, about 1000 bp of the 5′ *ldh* open reading frame including the ribosome binding site (rbs) of the *ldh* promoter were amplified with oligonucleotides Sg-ldh-up-F/Sg-ldh-up-R-ren, the renilla gene was amplified from plasmid pRL-TK (gift from Dr. Ralf Janknecht, University of Oklahoma Health Sciences Center) with oligonucleotides Sg-ren-F/Sg-ren-R. The Sg-ren-F primer introduced the *ldh* rbs on the 5′ end of the renilla gene. The *ermAM* gene cassette for selection of PCR product integration into the chromosome was amplified using oligonucleotides Sg-ren-erm-F/Sg-ren-erm-R and the *ldh* downstream fragment (about 1000 bp) was amplified with oligonucleotides Sg-ldh-down-F/Sg-ldh-down-R. Oligonucleotides were constructed with overlapping sequences as shown in Tab. 1. All four PCR amplicons were purified with the QIAGEN PCR purification kit and mixed in a 1∶1:1∶1 ratio. The mixture served as a template for a second round PCR with the appropriate up F and down R primers. The resulting PCR amplicons were transformed into DL1. Successful transformation was confirmed by testing several transformants for renilla reporter gene activity. The LytF and AtlS mutation were generated by transformation of chromosomal DNA from DL1-AtlS and SGH24, respectively. To assay for renilla activity, 100 µl of an exponentially growing culture was mixed with 0.5 µl of ViviRen™ Live Cell Substrate (Promega) from a 3.7 µg/1 µl stock solution. Bioluminescence was determined with a Modulus Luminometer (Turner BioSystems).

### RNA Isolation, cDNA Synthesis, and Real-time RT PCR

RNA was isolated using a Qiagen RNeasy kit, and cDNA was synthesized using qScript™ cDNA synthesis kit (Quanta Biosciences) according to the manufacturer’s protocol. Real-time RT PCR was performed to determine specific cDNA copies with the comparative threshold cycle (C_T_) method using a MyiQ single-color real-time PCR detection system (Bio-Rad) and PerfeCta™ SYBR^®^ Green SuperMix for iQ™ (Quanta Biosciences). Relative changes in cDNA copies representing differential gene expression were calculated using the ΔC_T_ method described previously (62). The 16S rRNA gene was used as the housekeeping reference gene using the 16S rRNA oligonucleotides described in Tab. 1.

### Determination of H_2_O_2_ Concentration

The concentration of H_2_O_2_ in liquid cultures was determined using a modification of the protocol described by Gilliland (12). Cell-free culture supernatants (40 µl) were mixed with 160 µl of freshly prepared 0.1 M sodium acetate (pH 4.5) containing 0.1 µg horseradish peroxidase (Thermo Scientific) and 10 µl of 1 mg/ml o-dianisidine (Alfa Aesar) in methanol. The reaction mixture was incubated at room temperature for 10 min and protected from light before A_415 nm_ was determined using a microplate reader (model 680; Bio-Rad). The concentration was calculated from a standard curve prepared in the same medium or buffer using a serial dilution of a commercial 30% H_2_O_2_ solution in MilliQ water. The concentration of the initial dilution was determined spectrophotometrically (ε_240_ = 43.6/M·cm) using a SmartSpec Plus UV-visible spectrophotometer (Bio-Rad) before each new experiment. The detectable range was 0.1 to 4.0 mM H_2_O_2_ in BHI.

### Observation of eDNA Release

The amount of eDNA in liquid cultures was measured directly by quantitative real-time PCR. A 2-µl aliquot of cell-free culture supernatant was mixed with 8 µl molecular-grade water (G-Biosciences), 12.5 µl PerfeCta™ SYBR^®^ Green SuperMix for iQ™ (Quanta Biosciences), 1.25 µl of primer 16S rRNA Sg-F, and 1.25 µl primer 16S rRNA Sg-R from a 10 mM stock solution. The PCR was performed in a MyiQ single-color real-time PCR detection system (Bio-Rad) and included one cycle of 95°C for 3 min, followed by 40 cycles of 95°C for 15 s and 55°C for 1 min. The DNA concentration was calculated based on average threshold cycle values against a 10-fold dilution series of purified DL1 genomic DNA in the same medium. The detectable range was 0.001 to 100 µg/ml DNA. The concentration of the standard was adjusted using a NanoDrop-1000 spectrophotometer (Thermo Scientific).

### Statistical Analysis

Statistical significance was calculated using a two-sided Student’s t-test and Quickcalcs online calculator (http://www.graphpad.com/quickcalcs). P values less than 0.05 were considered statistically significant.

## Results

### Influence of Aeration on Growth of Wild Type, AtlS, LytF and AtlS/LytF Mutants

**Figure 1:**
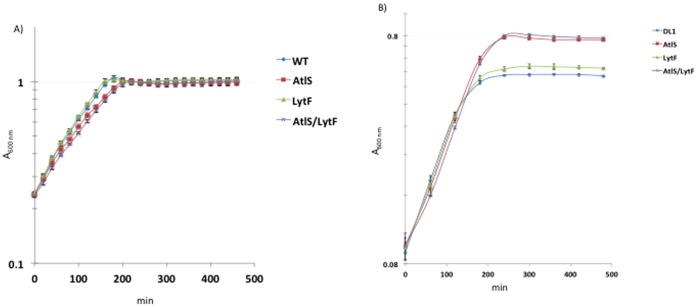
Growth curves of **S. gordonii** wild type, AtlS, LytF and AtlS/LytF mutants. A. Cells were grown as static cultures in ambient air at 37°C. Absorption was measured automatically using Bioscreen C every 20 min. B. Cells were grown aerobically as shaking cultures on a platform rocker at 37°C for maximal H2O2 production.

**Figure 2:**
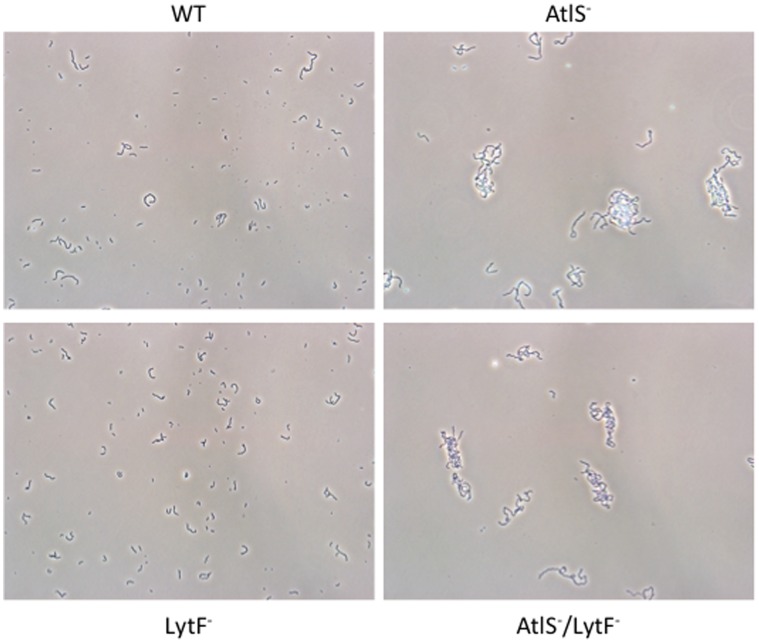
Elongated chain formation in the AtlS and AtlS/LytF mutant. Cells were grown to mid-logarithmic phase and phase contrast images taken at 400 fold magnification. The images are adjusted for contrast and brightness. Images were taken using an Olympus BX51 microscope, Olympus DP72 digital camera and cellSens 1.3 software. Shown is a representative of 2 independent experiments with similar outcome.

Previous experimental results linked the production of H_2_O_2_ to the release of eDNA in *S. gordonii* [12]. H_2_O_2_ is produced by the pyruvate oxidase (SpxB) under aerobic growth conditions and has a self-inhibitory effect on the producing species [12], [28]. To determine whether the introduction of the respective AtlS, LytF and AtlS/LytF mutations into *S. gordonii* causes any growth defects, growth was monitored under aerobic and static conditions, which has been shown to abolish H_2_O_2_ production [12]. During static growth, both the wild type and the LytF mutant showed nearly identical growth patterns (Fig. 1A). The AtlS and AtlS/LytF mutant strains showed a slightly reduced growth rate, but all four strains reached the same final bacterial density. In contrast, when cells were grown aerobically with maximal H_2_O_2_ production, the wild type and the LytF mutant strains reached stationary phase earlier than the AtlS and AtlS/LytF mutants, while the growth rate was identical (Fig. 1B). This observation is reminiscent of results obtained with aerobically grown streptococcal cells in the presence of catalase, which allows for increased cell density by avoiding the growth inhibitory effect of H_2_O_2_ [33]. The observed higher cell densities of the AtlS and AtlS/LytF mutants suggest an impaired H_2_O_2_ production. To exclude, however, that bacterial aggregation and therefore increased precipitation of bacterial aggregates is a result of the here observed growth phenotype cells were examined microscopically. Aerobically grown cells showed tangled and elongated streptococcal chains for the AtlS and AtlS/LytF mutants, while the wild type and LytF mutant grew in short chains (Fig. 2). This excludes a possible effect of cell aggregation on the bacterial density measurements. In conclusion, the observed growth phenotypes suggest that either the AtlS mutation confers some kind of resistance to the produced H_2_O_2_ or a lower H_2_O_2_ production in the AtlS mutants during aerobic growth allowing for higher final cell densities.

### Differential H_2_O_2_ Production by the Wild Type, AtlS, LytF and AtlS/LytF Mutants

**Figure 3:**
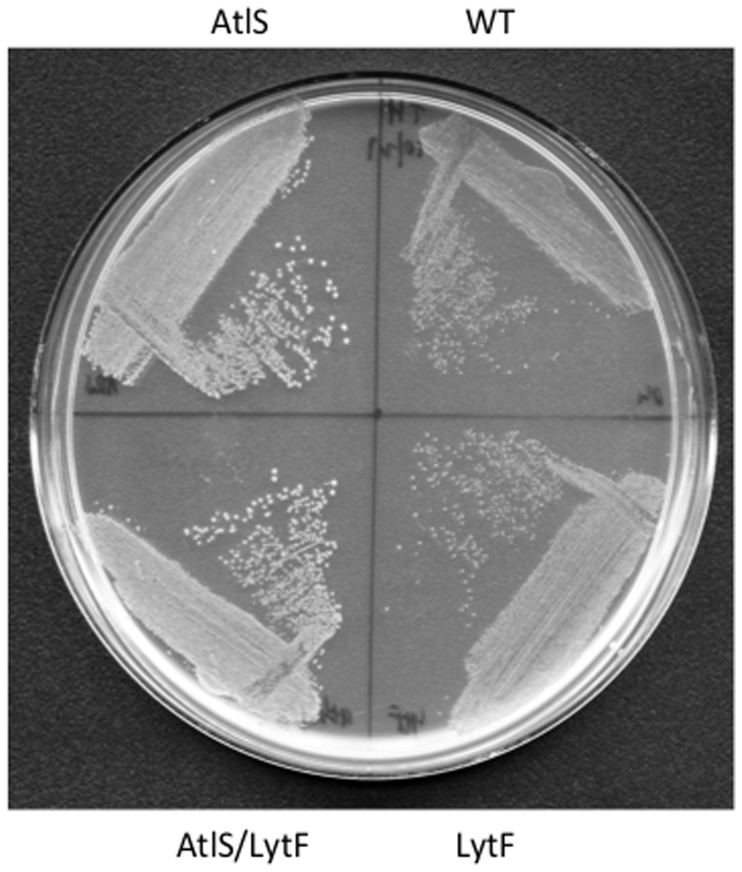
Oxygen dependent growth phenotype. Cells were grown under aerobic conditions on a TH plate overnight. The image is adjusted for contrast and brightness. Shown is a representative of 2 independent experiments with similar outcome.

**Figure 4:**
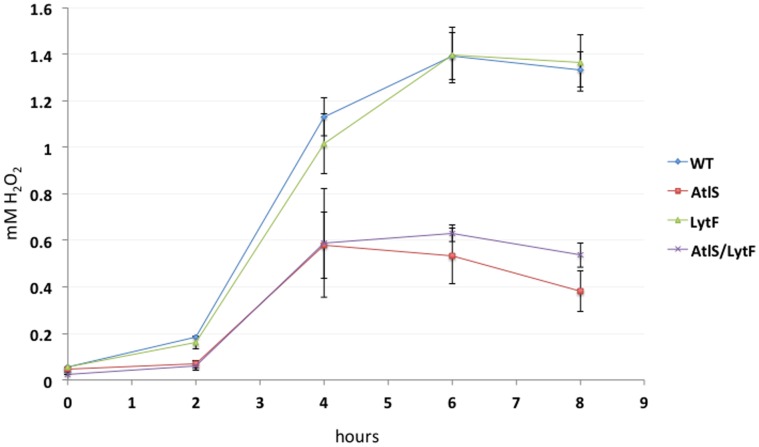
H_2_O_2_ production. H_2_O_2_ concentration was determined during growth under aerobic conditions. Error bars represent standard deviations from the mean (n = 3).

The observed difference in cell density of aerobically grown cultures could be the result of changes in H_2_O_2_ production of the respective mutant strains. Supporting a difference in H_2_O_2_ production, growth under aerobic conditions also leads to a clear difference in colony size between wild type, AtlS, LytF and AtlS/LytF mutants (Fig. 3). This was not observed during incubation in an anaerobic growth chamber (data not shown). The production of H_2_O_2_ was therefore monitored and no H_2_O_2_ production was observed during static growth as reported before [12]. During aerobic growth, both the wild type and the LytF mutant showed peak production of H_2_O_2_ during exponential growth, reaching about 1.4 mM when entering the stationary phase (Fig. 4), consistent with earlier reports [12]. In contrast, the AtlS and AtlS/LytF mutants only produced up to 0.6 mM when stationary phase was reached, about 40% of the wild type production capacity (Fig. 4). The net H_2_O_2_ production of the AtlS mutant was determined with cells grown aerobically to mid-exponential phase, showing about a 25% reduction when compared to the wild type (data not shown). These results suggest that the introduction of the AtlS mutation had a general effect on the H_2_O_2_ production capacity of strain DL1.

### Reduced Production of H_2_O_2_ is a Result of Decreased *spxB* Expression

**Figure 5:**
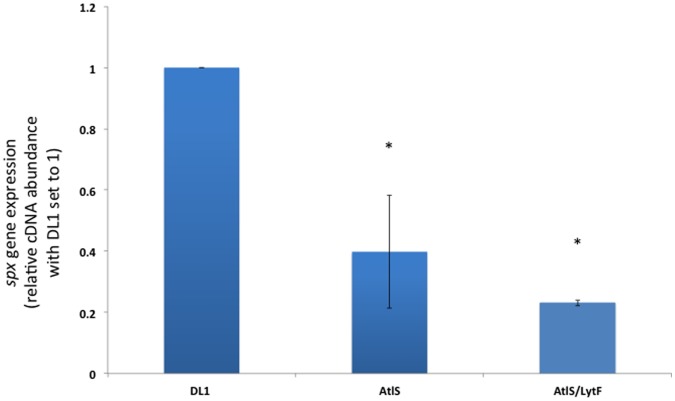
Expression of *spxB* under aerobic conditions. For comparative real-time RT PCR analysis, wild type, AtlS, and AtlS/LytF mutant cells were grown in BHI until mid-logarithmic phase. The expression level for wild type *spxB* was arbitrarily assigned a value of 1. The *gyrA* gene was used as the housekeeping reference gene. Error bars represent standard deviations from the mean (n = 3). Asterisks indicate statistically significant differences (p = 0.05) in *spxB* expression in comparison to the wild type.

H_2_O_2_ in *S. gordonii* originates mostly from the enzymatic activity of the pyruvate oxidase, SpxB (gene *spxB*) [34], [35]. SpxB catalyzes the conversion of pyruvate to acetyl phosphate, which subsequently is converted to acetate by acetate kinase [34]. The expression of the *spxB* gene was determined from cells grown aerobically to mid-exponential phase. In agreement with the observed lower H_2_O_2_ production shown in Fig. 4, cells carrying the AtlS mutation showed a statistically significant 2.5 to 4 fold lower *spxB* expression, respectively (Fig. 5).

### Release of eDNA

**Figure 6:**
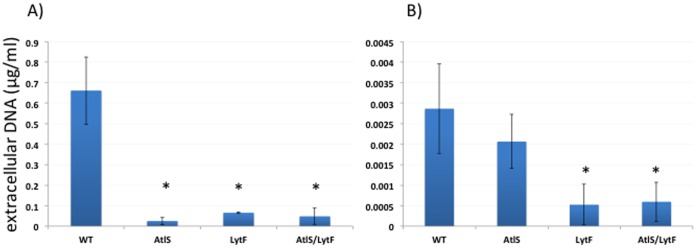
Release of eDNA during aerobic growth and as a result of H_2_O_2_ treatment. A. Wild type, AtlS, LytF and AtlS/Lytf mutant were grown until early stationary phase under aerobic conditions and the eDNA in the supernatant determined using Real-Time PCR. B. Cells were grown as static cultures under non-producing conditions and 2 mM H_2_O_2_ added after cells reached an A_600_ of 0.3. After further incubation of 5.5 hours, the eDNA concentration was determined in the supernatant using Real-Time PCR. Error bars represent standard deviations from the mean (n = 3). Asterisks indicate statistically significant differences (p = 0.05) in the eDNA concentration in comparison to the wild type.

AtlS and LytF have been implicated in the release of eDNA in *S. gordonii* [13], [29]. However, the eDNA release of the *lytF* mutant was not determined directly. To learn how the AtlS, LytF and AtlS/LytF mutation interferes with the ability of *S. gordonii* to release eDNA, cells were first grown under aerobic conditions to mid-exponential phase and the eDNA concentration measured in the supernatant. All mutant strains had a significant reduction in eDNA release, ranging from a 26-fold reduction for the AtlS mutant, 10-fold reduction for the LytF mutant and 14-fold reduction for the AtlS/LytF double mutant strain (Fig. 6A).

Since the release of eDNA in *S. gordonii* can be induced by H_2_O_2_ [12], it was important to determine if the eDNA release could still be induced in the mutant strains. Thus, cells were grown under static, non-producing conditions and 2 mM H_2_O_2_ was added to the cultures during mid-exponential growth. The cells were further incubated for 5.5 hours and the concentration of eDNA determined in the supernatant. As previously reported, the amount of eDNA released is lower when non-producing cells are challenged with H_2_O_2_ as compared to aerobically grown cells [12]. The AtlS mutant showed a reduced amount of released eDNA compared to the wild type, albeit not statistically significant (Fig. 6B). In contrast, the LytF and AtlS/Lytf mutant strains showed a statistically significant 5-fold reduction in the detectable eDNA (Fig. 6B), indicating that the LytF mutation interfered with the H_2_O_2_ induced eDNA release.

### Contribution of AtlS and LytF to *S. gordonii* Bacteriolysis

**Figure 7:**
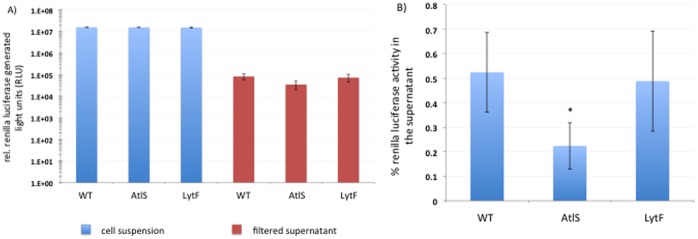
Detection of renilla reporter protein activity. A) Relative light units (RLUs) were measured with 100 µl cell suspension or filter-sterilized supernatants. B) Percentage of renilla luciferase activity in the supernatant normalized to the respective cellular renilla activity. Error bars represent standard deviations from the mean (n = 3). Asterisk indicates statistically significant difference in normalized renilla luciferase activity (p = 0.05).

To determine the contribution of AtlS and LytF to cell lysis, a renilla luciferase reporter protein strain was constructed [36]. This renilla luciferase (36 kDa) catalyzes the emission of visible light in the presence of oxygen and the substrate coelenterazine, and does not require any other co-factors [37]. In addition, the enzyme is highly stable over hours in supernatants [38]. The measurement of reporter protein activities in supernatants has been used before to determine bacterial lysis in connection with eDNA release [14], [15], [39]. The cells were grown as shaken cultures for maximal eDNA release and to avoid any oxygen limitation for the renilla enzyme. No difference in renilla activity was determined when cell suspensions were measured (Fig. 7A). The expression of the renilla reporter-fusion is therefore not influenced by the introduced mutations allowing for direct comparison of all strains. Next, the filter-sterilized supernatant was measured to determine the activity of the released renilla protein and normalized to the renillla activity of the respective cell suspension to determine the percentage of released renilla activity (Fig. 7B). The percentage of extracellular renilla activity was 0.52% ±0.2 for the wild type, 0.22% ±0.1 for the AtlS mutant and 0.49% ±0.2 for the LytF mutant. The difference between wild type and AtlS mutant is statistically significant.

## Discussion

The present report aims to better understand the mechanism of eDNA release in the oral commensal *S. gordonii*. We initially reported the observation of growth phase dependent eDNA release in *S. gordonii* and *Streptococcus sanguinis* [10]. The eDNA release was linked to the oxygen dependent metabolic enzyme pyruvate oxidase (SpxB). A deletion of the *spxB* gene diminished the release of eDNA in both organisms. Further examination revealed that the metabolic by-product of SpxB activity, H_2_O_2,_ is responsible for the observed eDNA release based on several lines of evidence: i) addition of the H_2_O_2_ degrading catalase or peroxidase decreased the released eDNA over 100 fold ([10] and unpublished result), ii) cells grown under anaerobic conditions producing no H_2_O_2_ failed to generate eDNA [12], [29], and iii) addition of physiological amounts of H_2_O_2_ to either anaerobically grown cells or to the *spxB* mutant triggered the release of eDNA [10], [12]. Interestingly, we did not observe an eDNA release associated cell density decline as reported for other species. Incubation for more than 90 hours under maximal H_2_O_2_ producing conditions did not result in changes to the final cell density determined spectrophotometrically. An autolysis assay known to induce bacteriolytic activity in other species failed to induce lysis in *S. gordonii* and *S. sanguinis* regardless if H_2_O_2_ was present or not [10]. Furthermore, no difference of the intracellular nucleoside triphosphate ATP was measured in the supernatant of the wild type *vs*. the SpxB mutant, further confirming that no general lysis is associated with eDNA release in both oral streptococci [10]. We concluded that both streptococcal species used in our studies did not lyse substantially under conditions known to cause lysis of other firmicutes, for example *E. faecalis* and *S. aureus* [10], [16], [40].

Our data, however, is in contrast to two recent observations implicating bacteriolytic activity to the release of eDNA in *S. gordonii* [13], [29]. First, the autolysin AtlS was identified to be essential for autolysis in *S. gordonii*. A knock-out mutant did not release eDNA at all [29]. Second, the muralytic LytF protein seemed to be involved in the competence dependent release of eDNA [13]. LytF is a functional analog to the well-characterized *Streptococcus pneumoniae* murein hydrolase CbpD [41]. Both AtlS and LytF have proven murein-hydrolyzing activity in *in vitro* zymorgraphic assays [13], [29]. LytF however, is only active during competence development and its muralytic activity seems to be limited to a sub-fraction of cells [13]. The here used DL1 wild type and AtlS mutant were identical to the one used in [29]; however, the LytF mutation, originally in another *S. gordonii* strain, NCTC 7865, was transferred to the DL1 strain. The original AtlS study used 1/4-strength BHI medium supplemented with 10 mM sucrose [29]. Since sucrose causes carbon catabolite dependent repression of *spxB* expression [42], our study used full strength BHI without any added carbohydrates.

Quantitation of the released eDNA in the wild type, AtlS, LytF and AtlS/LytF mutant strains showed an obvious reduction in the produced eDNA in the mutants, ranging from 10 to 26 fold less eDNA in the supernatant compared to the wild type. This initially supported an involvement of AtlS and LytF in the eDNA release process. Further investigation, however, of the H_2_O_2_ production pattern showed that the AtlS mutation affected the concentration of H_2_O_2_ during the exponential growth phase leading to a twofold lower end concentration of H_2_O_2_ in the supernatant. The reduction was the result of a decreased *spxB* expression in the mutants carrying the AtlS mutation. This has a profound effect on the eDNA release, since our earlier data showed that a threshold H_2_O_2_ concentration is required for eDNA release [12]. Detectable amounts of eDNA were only released when the H_2_O_2_ concentration reached amounts higher then 0.6 mM, with the maximal release of eDNA at concentrations around 1 to 2 mM [12]. We suspected that the AtlS mutant did not produce sufficient amounts of H_2_O_2_ to trigger the eDNA release process. To confirm this, we added H_2_O_2_ to exponentially growing AtlS mutant cells under non eDNA releasing conditions and confirmed that the AtlS mutant was still inducible for eDNA production comparable to wild type amounts. Our new results argue against an involvement of AtlS in the H_2_O_2_ dependent eDNA release process, however, the effect of AtlS on H_2_O_2_ production and *spxB* expression requires further investigation to understand the causal relationship between these observed phenotypes.

The eDNA release was not associated with a detectable cell lysis as reported in this study and earlier [10], suggesting that only a subpopulation of cells lyse and releases eDNA or that the actual release process is not caused by complete bacterial lysis, leaving the cell envelope mostly intact. The study by Berg *et al.* demonstrated that most of the *S. gordonii* cells are not affected by the muralytic activity of LytF and that only a fraction of the cell population is lysed. To determine the contribution of AtlS and LytF to cell lysis, we measured the release of a reporter protein into the medium during aerobic growth. This growth condition promoted the highest amount of eDNA release [10], [12], [29]. Interestingly, the activity measured in the supernatant was comparable between the wild type, the AtlS mutant and the LytF mutant and about 1000 fold over growth medium background. After calculating the percentage of renilla activity in the supernatant a two-fold significant reduction of activity for the AtlS mutant was detectable with 0.52% ±0.2 for the wild type and 0.22% ±0.1 for the AtlS mutant. The LytF mutant was comparable to the wild type with 0.49% ±0.2, respectively. For comparison, the CbpD mutant encoding the functional LytF analog of *S. pneumoniae* had a 80-fold reduction in a *β*-galactosidase release assay, when compared to the wild type [43]. The *S. aureus cidA* mutant encoding the murein hydrolase regulator involved in DNA release had a 10-fold difference in the *β*-galactosidase release assay, when compared to the wild type [15]. The low extracellular activity of the renilla enzyme of the wild type, AtlS and LytF mutant suggests that complete cell lysis is not a major factor in the eDNA release process.

Our data, however, suggests that LytF is the responsible enzyme for the here-observed eDNA release, since the LytF mutant strain is no longer inducible for eDNA release. An involvement of LytF also makes sense considering that its gene is part of the competence system. The *S. gordonii* competence system is induced under H_2_O_2_ producing conditions [12]. Competence in general is considered a major stress response allowing for the uptake of environmental DNA for repair and recombination [44]. *S. gordonii* appears to release eDNA as part of competence development under stress situations. Further research is required to understand to what extend LytF lyses cells and releases eDNA, but not cellular content such as enzymes and ATP as shown before.
